# Brucellosis caused by the wood rat pathogen *Brucella neotomae:* two case reports

**DOI:** 10.1186/s13256-017-1496-8

**Published:** 2017-12-19

**Authors:** Juan M. Villalobos-Vindas, Ernesto Amuy, Elías Barquero-Calvo, Norman Rojas, Carlos Chacón-Díaz, Esteban Chaves-Olarte, Caterina Guzman-Verri, Edgardo Moreno

**Affiliations:** 1 Caja Costarricense de Seguro Social, San José, Costa Rica; 2 Programa de Investigación en Enfermedades Tropicales, Universidad Nacional, Heredia, Costa Rica; 3 Centro de Investigación en Enfermedades Tropicales, Universidad de Costa Rica, Heredia, Costa Rica; 4 Instituto Clodomiro Picado, Universidad de Costa Rica, San José, Costa Rica

**Keywords:** *Brucella neotomae*, Brucellosis, Neurobrucellosis, *Brucella*, Zoonosis

## Abstract

**Background:**

Brucellosis is a chronic bacterial disease caused by members of the genus *Brucella*. Among the classical species stands *Brucella neotomae*, until now, a pathogen limited to wood rats. However, we have identified two brucellosis human cases caused by *B. neotomae*, demonstrating that this species has zoonotic potential.

**Cases presentation:**

Within almost 4 years of each other, a 64-year-old Costa Rican white Hispanic man and a 51-year-old Costa Rican white Hispanic man required medical care at public hospitals of Costa Rica. Their hematological and biochemical parameters were within normal limits. No adenopathies or visceral abnormalities were found. Both patients showed intermittent fever, disorientation, and general malaise and a positive Rose Bengal test compatible with *Brucella* infection. Blood and cerebrospinal fluid cultures rendered Gram-negative coccobacilli identified by genomic analysis as *B. neotomae*. After antibiotic treatment, the patients recovered with normal mental activities.

**Conclusions:**

This is the first report describing in detail the clinical disease caused by *B. neotomae* in two unrelated patients*.* In spite of previous claims, this bacterium keeps zoonotic potential. Proposals to generate vaccines by using *B. neotomae* as an immunogen must be reexamined and countries housing the natural reservoir must consider the zoonotic risk.

## Background

Brucellosis is an infectious bacterial disease of animals and a relevant zoonosis [1]. Four *Brucella* species have been reported to be the main cause of human infections: *Brucella abortus*, *Brucella melitensis*, *Brucella suis*, and *Brucella canis* [1]. In addition, rare *Brucella* isolates have been reported in human cases, including some strains similar to those isolated from marine mammals [2–4]. In all these cases the identification of *Brucella* organisms has relied on molecular methods, with no epidemiological information that could explain the source for transmission to humans.

*Brucella neotomae*, first isolated in 1957 in desert wood rats of the genus *Neotomae* in the United States of America (USA) [5], has been considered for 60 years a non-zoonotic bacterium and therefore of no risk to humans. However, *B. neotomae* has been isolated in the organs of wood rats and has been shown to display pathogenicity for mice [6]. Moreover, whole genome analysis of *B. neotomae* has revealed that this bacterium possesses the same virulence arsenal as the classic zoonotic brucellae [7]. Therefore, its potential as a human pathogen cannot be ruled out a priori. Here we describe two clinical cases of brucellosis caused by *B. neotomae* and discuss the medical and epidemiological implications of our findings.

## Case presentation

### Case 1

A 64-year-old Costa Rican white Hispanic man, an inhabitant from the Central Valley of Costa Rica (1000 m altitude), presented to a Costa Rican Social Security Hospital. He was hypertensive. On arrival, he presented a lung infection and a stroke with an intraparenchymal hemorrhage of the right basal ganglia with extension to the ipsilateral lateral ventricle, as demonstrated by computed axial tomography. He was treated with penicillin G (10^6^ units/intramuscular/7 days) and received medical intervention for 7 days. After this period he recovered with no recurrence of his illness.

He presented at the hospital again, 13 months after this event, with left hemiparesis, headache, disorientation, dysarthria, lethargy, and intermittent fever. He did not respond to questions and did not follow instructions. He was kept at the hospital for examination, observation, and treatment. His hematological and biochemical parameters were within normal ranges, including leukocyte counts and cell morphology. A C-reactive protein test was negative. An abdominal ultrasound did not demonstrate alterations and other tests revealed normal size and function of his liver and spleen. Diagnostic findings by an echocardiogram did not reveal any vegetation or other sequelae of infectious endocarditis. Computed axial tomography showed sequelae related to the stroke he had 1 year before.

**Fig. 1:**
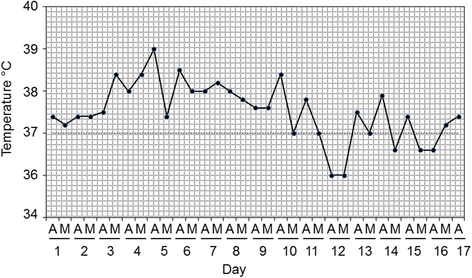
Clinical chart displaying the (undulant) intermittent fever experienced during hospitalization by patient of case 1. Temperature was taken during the morning and afternoon with 12 hours difference. *A* afternoon, *M* morning

After hospitalization, he had some improvement, but the intermittent fever remained (Fig. 1). After 12 days, a blood culture for the presence of bacteria was performed in blood agar, with negative results after 3 days of incubation. At the fourth day, a second blood culture was taken and cerebrospinal fluid extracted, analyzed, and cultured in blood agar. Concomitantly, he received treatment with 1 gram of cefotaxime by an intravenous route every 8 hours. The cerebrospinal fluid was cloudy with protein levels of 220 mg/dL, 20 erythrocytes/mm^3^, and 222 leukocytes/mm^3^ with 94% lymphocytes. The first blood culture was kept up to 1 week, with negative results. He showed positive agglutination in Rose Bengal test for brucellosis. After 3 days of incubation of the second blood culture, Gram-negative coccobacilli compatible with *Brucella* were isolated. Likewise, Gram-negative coccobacilli similar to that from the blood culture were also isolated in blood agar from the cerebrospinal fluid, after 3 days. The isolates were highly sensitive to a panel of antibiotics used to treat brucellosis [8]. The cefotaxime treatment was interrupted and a combination of doxycycline (100 mg/12 hours by the oral route) and streptomycin (750 mg/24 hours, by the intramuscular route) was given for 12 and 4 weeks, respectively. After antibiotic treatment, he recovered with no fever and with normal mental activities, but remained with slight left hemiparesis sequelae.

In the course of this study, no species was assigned for the isolated *Brucella* species. Five years later, the bacterium was identified as *B. neotomae* by multiple-locus variable number tandem repeat analysis of 16 sequences (MLVA16) and whole genome sequencing [7].

### Case 2

A 51-year-old Costa Rican white Hispanic man from Puntarenas, East Pacific coast of Costa Rica, presented to the local Social Security Hospital 3 years and 11 months after Case 1. He presented a recurrent headache, disorientation, general muscle and joint pain, weight loss, cough, anorexia, and intermittent nocturnal fever of 3 weeks of evolution. Since he had a previous clinical history of dengue fever and lived in an endemic dengue region on the Pacific coast of Costa Rica, he was hospitalized as a possible case of dengue. On general examination, he did not present rash, adenopathies, abdominal pain, or visceral enlargement. Blood tests showed normal leukocyte counts. With the exception of a mild thrombocytopenia, a hemogram was within normal parameters. A C-reactive protein test was negative. The results of a differential molecular diagnosis by polymerase chain reaction (PCR) for dengue, toxoplasmosis, cytomegalovirus, malaria, and Epstein–Barr virus were all negative. He showed positive agglutination in Rose Bengal test for brucellosis. Gram-negative coccobacilli compatible with *Brucella* species were recovered from blood after 3 days of bacteriological culture in blood agar. The isolate was highly sensitive to a panel of antibiotics used to treat brucellosis [8]. Following this, he was treated with doxycycline (100 mg/12 hours by the oral route) and rifampin (900 mg/day orally) for 45 days. For the next days he showed considerable health improvement and after 6 days of hospitalization he was released. A follow up in local medical clinics was established. After the end of the antibiotic treatment, the Rose Bengal test became negative and he recovered with no sequelae.

Initially, the isolated etiological agent was misidentified as *B. abortus* by bacteriological and biochemical tests. However, 2 years later, when the isolates were analyzed by MLVA16 and whole genome sequencing, it became evident that the bacterial strains belonged to *B. neotomae* species [7].

## Discussion

Here, we have described in detail two human clinical cases caused by *B. neotomae*. In a previous work, we reported the phylogenetic and genomic characteristics of the *B. neotomae* strains isolated in these two human cases [7]. This is relevant, considering that the identification of *B. neotomae* is not straightforward and to distinguish it from other *Brucella* species by common bacteriological techniques is difficult [9]. Several years after the isolation of the etiological agents causing brucellosis in these two patients, the *Brucella* species were unambiguously identify following MLVA16 and whole genome sequence analysis [6]. Therefore, these techniques are valuable tools for the recognition of non-conventional *Brucella* strains.

The outcomes of the disease in the two *B. neotomae* clinical cases described here, do not depart from other classical brucellosis cases reported elsewhere [1, 10–12]. In fact, the course of zoonotic brucellosis, in general, is chronic with intermittent fever and a broad range of non-pathognomonic symptoms. In some instances, the bacterium is also capable of crossing the blood–brain barrier and invading the brain, causing neurobrucellosis [13], as in one of the cases presented here.

From the clinical perspective and at a glance, brucellosis is of difficult diagnosis and commonly is confused with other diseases that also cause intermittent fever. In fact, in contrast to other bacterial diseases, the course of brucellosis might not present endotoxicity signs, increase in proteins related to inflammatory processes and coagulopathies, or significant blood leukocyte changes [10–12]. However, in long-lasting cases, absolute neutropenia, thrombocytopenia, and increase of some proinflammatory proteins may be observed in approximately one third of the cases [10–12].

In tropical countries such as Costa Rica the differential diagnosis has to be carried out with dengue, chikungunya, Zika virus, malaria, and some viral infections. Moreover, classical diagnostic tests such as agglutination may be negative in a number of brucellosis cases, precluding a straightforward diagnosis [14]. For this, repeated serological testing and blood cultures and, when required, cerebrospinal fluid cultures (in the case of neurological disorders) are recommended before antibiotic treatment. Once the diagnosis has been established, treatment with a combination of two antibiotics for several weeks becomes mandatory. Fortunately, antibiotic resistance has seldom been reported in brucellosis [1] and up to now all strains, including *B. neotomae* (as demonstrated here) are susceptible to aminoglycoside and antibiotics of the tetracycline and rifampicin class.

It is intriguing how these two persons acquired *B. neotomae* infections within almost 4 years of each other and living in two different regions of the country. From the epidemiological perspective it is worth noting that in Costa Rica there are no rats of the genus *Neotomae*, although there are other Neotominae species such as those of the genus *Reithrodontomys* which are endemic [15]. Whether these rat species could serve as reservoirs for *B. neotomae* remains unknown. In any case, it is clear that human brucellosis is not limited to the so-called “classical” zoonotic *Brucella* species, and other members of the genus may also keep this potential. This is relevant, considering that countries such as USA and Canada (that have eradicated zoonotic *Brucella* species from cows, sheep, pigs, and goats) may still harbor *B. neotomae* in wood rats. Moreover, works devoted to generate vaccines by using *B. neotomae* as an immunogen [16, 17] must be reexamined.

## Conclusions

Physicians should consider brucellosis caused by *B. neotomae* as one of the agents for human disease. Even in those areas where *Brucella* organisms have been eradicated from domestic animals, *B. neotomae* may still be a zoonotic risk. The fact that *B. neotomae* is capable of invading the brain indicates the robust pathogenic potential of *B. neotomae*. Differential diagnosis with other diseases that present with intermittent fever is necessary, whether they are in endemic or non-endemic areas.

## Abbreviation

MLVA16: Multiple-locus variable number tandem repeat analysis of 16 sequences
